# The Nature and Measure of Critical Thinking: The PACIER Framework and Assessment

**DOI:** 10.3390/jintelligence13090113

**Published:** 2025-09-02

**Authors:** Hyo Jeong Shin, Seewoo Li, Ji Hoon Ryoo, Alina von Davier, Todd Lubart, Salah Khalil

**Affiliations:** 1 College of Humanities and Graduate School of Education, Sogang University, Seoul 04107, Republic of Korea; 2 Department of Education, School of Education & Information Studies, University of California Los Angeles, Los Angeles, CA 90095, USA; seewooli@g.ucla.edu; 3 Department of Education, College of Educational Sciences, Yonsei University, Seoul 03722, Republic of Korea; ryoox001@yonsei.ac.kr; 4 EdAstra Tech LLC, Newton, MA 02458, USA; avondavier@edastratech.com; 5 Laboratoire de Psychologie et d’Ergonomie Appliquée (LaPEA), Université Paris Cité and Univ Gustave Eiffel, F-92100 Boulogne-Billancourt, France; todd.lubart@u-paris.fr; 6 Macat, London SW64LZ, UK; salah@macat.com

**Keywords:** critical thinking, computer-based assessment, item response theory, 21st-century skills

## Abstract

Based on the PACIER model of critical thinking, involving six facets for critical thinking (Problem solving, Analysis, Creative thinking, Interpretation, Evaluation, Reasoning), the empirical results of a new computer-based assessment (PACIER Critical Thinking Assessment) are presented. The data is based on a study of 700 middle school 11-year-old students in the United Arab Emirates, who were tested five times during a school year. In the assessment framework, test items are described, and psychometric results indicate that the PACIER Critical Thinking Assessment exhibits acceptable reliability and validity. Its use for measuring progress in educational programs to foster critical thinking is discussed.

## 1. Introduction

Educators, researchers, policymakers, and employers acknowledge the need to train people to think critically for success in personal life decisions, workplace performance, and as citizens involved in societal debates and decisions (Halpern and Dunn 2021). For decades, research on critical thinking has sought to define it, understand its components, develop and implement pedagogical activities to foster it, and design assessments to measure success (Facione 1990a; Yücel 2025). Early work, in the first half of the 20th century, drew on logic and philosophy, emphasizing reflection and reasoned judgment (see (Dewey 1910)). Mid-20th-century work framed critical thinking as a set of cognitive skills including inference and evaluation (Ennis 1996). From the 1980s, dispositions or preferences to engage in critical thinking were included (Facione 1990a; Ennis 2011). In the last two decades, the concept of critical thinking as a partially domain-specific skill set developed. Furthermore, notable contributions by the OECD led to rubrics to assess critical thinking, with benchmarked educational activities to promote it (Vincent-Lancrin et al. 2019). There were also recent trends to connect critical thinking with other 21st-century skills, including creativity, collaboration, and communication. This article focuses on examining the psychometric qualities of a new computer-based assessment based on the PACIER model to support educators as they seek to develop critical thinking and measure progress to foster tomorrow’s critical thinkers (Dwyer 2023).

How to implement critical thinking in education is a field of massive inquiry. A bibliometric analysis of more than 6000 articles published between 2005 and 2024 found a consistent increase in research on critical thinking in education, with a notable spike in 2023 (Yücel 2025). There is additionally a growing focus in the literature on the intersection between emerging technologies such as generative AI and critical thinking in education (Ahmad et al. 2023).

From news and social media algorithms that promote echo chambers and reinforce confirmation bias to the rapid rise in AI tools resulting in possible deep fakes or misinformation, critical thinking skills are increasingly important. However, today’s mass adoption of AI poses danger and opportunity for critical thinking skills in educational settings depending on how students choose to interact with AI. A study of 285 university students in China and Pakistan found that using AI in education increases the loss of human decision-making capabilities and makes users lazy by performing and automating the work, with 68.9% of laziness and 27.7% loss of decision-making attributed to the impact of AI (Ahmad et al. 2023). As learning and research environments increasingly integrate AI dialogue systems, there is a risk that users unquestioningly accept recommendations, reducing critical and analytical thinking skills, diminishing judgment associated with critical information analysis, and leading to decisions that cause errors in task performance (Zhai et al. 2024).

Additionally, in the emerging global work landscape, jobs are more rapidly evolving than ever, aligned with an accelerating pace of technological change. Four out of five executives believe that employees’ roles and skills will change as a result of generative AI (IBM 2023). Employers anticipate that 39% of essential worker skills will change by 2030, with an increasing focus on programs to continuously learn, upskill, and reskill. Analytical thinking, however, remains in the top five skills that employers consider as core to their workforce (World Economic Forum 2025). On average, 87% of surveyed executives envision roles to be augmented rather than replaced by generative AI (IBM 2023). As AI grows its repository of human knowledge, serves it to us on demand, and creates content, the opportunity will be to harness AI by applying critical thinking to boost human intelligence. There is a shift underway from knowledge-based, functionally siloed jobs to skills-based work in jobs (Cantrell et al. 2022). In this emerging world, skills must be enduring and transferable as work needs and opportunities rapidly evolve. Among the skills that are most often recognized as important for the future, critical thinking figures systematically (Care et al. 2012).

## 2. Thinking Critically About Critical Thinking

In a Monty Python comedy skit, a man walks into an education clinic and pays for five minutes of argumentation. He asks the teacher if he is in the correct room for an argument. The instructor insists on having already answered this question. A tit-for-tat ensues: “No, you haven’t.” “Yes, I have.” Finally, the conclusion is that this is simply a contradiction and not an argument, which takes place in another room. However, the challenge of defining an argument has been a matter of debate in the scientific literature, and the endeavor to define critical thinking has itself engaged critical thinking about how to research, measure, and teach it.

To date, there is no single, standardized definition. However, there is precedent for establishing a composite list of attributes that are foundational to evolving constructs on critical thinking. This list includes reflective thought, skepticism, self-regulatory judgment, analysis, evaluation, inference, and argument-based reasoning. Bloom (1956) developed a taxonomy of educational objectives that identified six major categories arranged as a hierarchy from the lowest to highest order, with a stated goal of stimulating thought about educational problems. The taxonomy identified evaluation as the highest-order skill, followed by synthesis and analysis (see also (Anderson and Krathwohl 2001)). In general, recent critical thinking definitions highlight goal-directed judgment and evidence-based reasoning. There is continuing debate on the importance of dispositions and traits, creativity, and the domain specificity or narrow vs. broad nature of critical thinking.

The American Philosophical Association convened a panel of experts to develop a consensus definition of critical thinking for the purpose of educational assessment and instruction (Facione 1990a). The report identified six skills: (1) interpretation, (2) analysis, (3) evaluation, (4) inference, (5) explanation, and (6) self-regulation. The panel associated sub-skills with each skill. Further work has addressed a dispositional dimension of critical thinking, stating that affective dispositions are necessary for the foundation and development of critical thinking skills (Ennis 1996).

## 3. Common Components Across Critical Thinking Assessments

Researchers have proposed a number of frameworks that break down critical thinking into component skills. A review of the literature suggests that among the multiple ways of framing critical thinking, there are some overlapping elements that are foundational to its measurement. For example, aligned with Bloom’s identification of *evaluation*, Halpern asserts that the word “critical” in critical thinking indicates a noteworthy evaluation component by which we evaluate the outcomes of our thinking, such as the strength of a decision or solution (Halpern 2014). In terms of assessment tools, for example, the *Watson-Glaser Critical Thinking Appraisal* tool (WGCTA, (Pearson 2020)) includes evaluation of arguments, and the *California Critical Thinking Skills Test* (CCTST, (Facione 1990b)) includes evaluation (Liu et al. 2018).

*Reasoning* is another ability measured in multiple critical thinking assessments, including the CCTST, *Collegiate Learning Assessment+* (CLA+, (Aloisi and Callaghan 2018)) and the *Halpern Critical Thinking Assessment* (HCTA, (Halpern 2010)). Still, there are differences. For example, the CCTST assesses overall reasoning skills. CLA+ measures scientific and quantitative reasoning. The HCTA measures verbal reasoning as a sub-skill, and the Ennis-Weir Critical Thinking Essay Test (Ennis and Weir 1985) measures the ability to offer good reasons (Liu et al. 2018).

*Analysis* is included in CCTST, CLA+ (which combines it with problem-solving), and HCTA (which combines argument and analysis skills), for example (Liu et al. 2018). Other common topics covered to varying degrees in critical thinking assessments as definitional elements are the ability to recognize assumptions, make inferences, deduce and induce (relates to reasoning), and engage in interpretation.

The measurement of subskills is an important consideration in test design, particularly as it leads to the development of instructional and training modules to master critical thinking (Bouckaert 2023). Although critical thinking lacks a universally agreed definition, the result of its attributes is logic-based thought that involves detecting biases, whether in solving problems, developing educated opinions, making sound decisions, or taking action. Ennis (2011), a pioneer in research on critical thinking, simply defines critical thinking as “reasonable and reflective thinking focused on deciding what to believe or do”. Thornhill-Miller et al. (2023) consider the following definition of critical thinking to be helpful for being specific, straightforward, and unequivocal in its implications for the education and evaluation of critical thinking skills: “the capacity of assessing the epistemic quality of available information and—as a consequence of this assessment—of calibrating one’s confidence in order to act upon such information”. Critical thinking provides a foundational set of real-life skills with practical applications in a rapidly changing world. It helps us to learn, work, and live better as we minimize errors and maximize success through adaptability and a greater capacity to confront emerging challenges.

## 4. A Set of Skills, Measurable and Teachable

Today there is broad acceptance that critical thinking is an acquired, teachable capacity rather than an innate ability (e.g., (Facione 1990a; Halpern 2014)). The OECD argues for embedding critical thinking in all subjects in school curricula not only to improve the skill but also to enrich understanding of the subject material. This gives students repeated opportunities to learn and practice the skills within the context of subjects they enjoy (Vincent-Lancrin et al. 2019; Vincent-Lancrin 2023). However, assessment of critical thinking is rarely embedded in regular school or university activities yet (Butler 2024).

To mainstream critical thinking skills, educators must be able to assess it, establish a baseline, and measure the outcomes of training. School systems seek viable ways to assess critical thinking skills to establish clear outcomes for success and then to link testing to systematic training for integrating skills into their curricula.

## 5. The PACIER Model

In a Cambridge University survey of 122 academics, respondents described their conception of critical thinking in terms of a range of thinking skills by ranking the importance of each skill to their subject discipline (Baker and Bellaera 2015; Bellaera et al. 2021). The survey identified three skills—analysis, evaluation, and interpretation—as the most highly valued, and it revealed additional thinking skills as fundamental. This resulted in a synthesis of skills that together describe critical thinking as an umbrella term (Dash 2019).

The PACIER model, developed by Macat (www.macat.com, accessed on 1 May 2025) in collaboration with the University of Cambridge, highlights six skills that collectively describe the propensity to think critically. When these related but somewhat distinct skills are combined, they become facets of critical thinking as a greater whole. Each letter of the acronym (PACIER) represents one skill, and each skill breaks down into four sub-skills.

Table 1 specifies each of the six skills and lists associated sub-skills. With respect to the American Philosophical Association consensual definition, self-regulation relates to problem solving, analysis, interpretation, and evaluation to the same components in PACIER and inference to reasoning. The creative thinking component in PACIER does not have a direct referent in the APA definition.

## 6. Particular Aspects of the PACIER Model

The following are some aspects of the PACIER model which distinguish it from other models.

1. Includes creative thinking as a facet of critical thinking

Critical thinking and creative thinking are often conceived as distinct processes, each playing a different role depending on the context of a situation; usually creative thinking applies to idea generation, and critical thinking applies to evaluating and implementing ideas (Wechsler et al. 2018). However, creative thinking supports critical thinking as it allows individuals to generate test cases, hypotheses that may prove an argument as flawed (Guignard and Lubart 2017). Well-known models and frameworks, such as Ennis (2011), Facione (1990a), or Paul and Elder (2014), do not include creativity. Standardized assessments rarely consider creativity as a distinct attribute of critical thinking. The PACIER Critical Thinking Assessment developed by Macat includes creativity as a key facet of critical thinking (Bouckaert 2023; Dwyer et al. 2025).

2. Includes domain-agnostic and domain-specific assessment

Critical thinking assessment models vary, with some domain-agnostic (e.g., (Ennis 2011; Paul and Elder 2014; Anderson and Krathwohl 2001)) and others domain-specific (e.g., medical and nursing), which measure context-specific aspects of critical thinking (Bouckaert 2023). Work on 21st-century competencies suggests that domain-specific knowledge is essential; domain specificity supports the practical application of the model by placing critical thinking skills in context based on real-world scenarios. The PACIER model supports the generation of both domain-agnostic and domain-specific assessments for each of the skills. Domain-agnostic items apply PACIER skills to general life contexts, whereas domain-specific items involve in-depth knowledge with domain-relevant criteria to score the quality of critical thinking responses.

3. Integrates real-world scenarios

In her review of critical thinking assessments, Butler (2024) notes that few assessments demonstrate that their scores are predictive of everyday contextualized behavior to support the practical application of critical thinking skills (Saiz and Rivas 2023). The PACIER framework moves toward this goal by linking to contextualized, domain-specific assessments and basing domain-agnostic items on real-world everyday life situations.

4. Takes a holistic approach to testing and training

By breaking down critical thinking into six components at the level of skills and more fine-grained sub-skills, the PACIER framework served as the basis of a training program to foster critical thinking. The PACIER Critical Thinking Assessment provides a global, wide-range assessment because it covers the broad set of facets that contribute to critical thinking. The PACIER test also allows the efficacy of training programs (the PACIER training program and other programs) to be examined in terms of the effects of training on each subskill.

## 7. Measurement of Critical Thinking Skills

In accordance with the framework, the PACIER Critical Thinking Assessment was developed with the objective of measuring all six domains through the implementation of computer-based assessments (CBA). The generation of assessment items was conducted in two ways: either by human experts alone or by a combination of human experts and generative artificial intelligence (AI), followed by a rigorous review process involving human experts.

**Figure 1:**
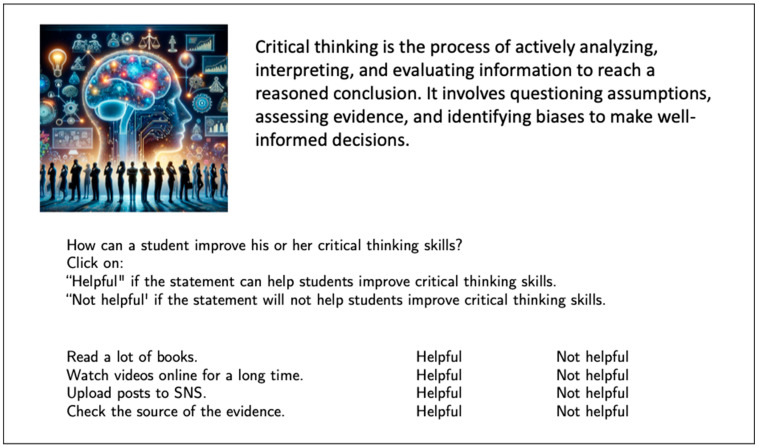
Example PACIER Critical Thinking Assessment Item (Multiple-Choice). SNS: social network systems.

In the pilot test, there were two main types of items in the PACIER Critical Thinking Assessment: multiple-choice items, where students were asked to select one of several (usually two) response options, and fill-in-the-blank items, where students were asked to place multiple words in the correct blanks. An illustrative item for the multiple-choice item is presented in Figure 1, which comprises a passage, an associated image, an instruction, and four statements for students to select from. Table 2 illustrates one sample item per domain according to the PACIER framework.

The students’ responses were scored based on the number of correct choices they made. Consequently, each item was assigned a score ranging from 0 to 4.

## 8. Pilot Study Design and Methodology

A pilot study was conducted to assess the psychometric properties of the PACIER Critical Thinking Assessment, focusing on reliability, validity, and item quality. The analysis of the pilot study aims to ensure that the assessment measures critical thinking skills as intended and to provide quantitative justification for the PACIER framework as the conceptual basis for these skills. The following section demonstrates the implementation of the assessment, providing examples of item bank construction, test design, and the use of a psychometric model.

### 8.1. Participants

During the 2023–2024 school year, three schools offering International Baccalaureate (IB) programs, located in the United Arab Emirates, participated in the pilot. Throughout the year, five computer-based assessments were administered to Year 6 (11-year-olds) students. There were approximately 700 students involved, and the number of participating students per school per assessment is shown in Table 3.

The school governing board approved the educational program with periodic testing as part of the school curriculum. The anonymous test scores were used for research purposes. All participants had parental consent. The Helsinki (World Medical Association 2013) guidelines for ethical research were followed.

### 8.2. Test

In order to assess students’ performance in critical thinking skills and evaluate the psychometric properties of the PACIER Critical Thinking Assessment, we assembled multiple test forms, administered different test forms to schools per assessment, and analyzed the test results using the measurement models based on the item response theory (IRT).

More specifically, out of a total of 96 items written in English, we assigned 12 items (2 items in each PACIER domain) to one cluster (namely, A, B, C, …, to H) and paired two clusters together as a single test form. In other words, each student took one test form consisting of 24 items, with 4 items in each of the six PACIER domains, for a given assessment. Students were given one hour to complete the test.

### 8.3. Assessment Design

To make the resulting test scores comparable across five assessments using the IRT concurrent calibration, we deliberately overlapped a few clusters across the five assessments with minimal overlap of item exposure. For example, the following design in Table 4 was administered in one of the schools. During the first assessment, clusters of A and B were administered in this school, while clusters of D and E were administered during the second assessment. Note that cluster A was repeated along with the cluster of G during the fourth assessment so that the first and fourth assessments could be linked through common items in cluster A. Although Table 4 does not appear to link some of the clusters, all clusters are fully linked when all 3 schools are included in the concurrent IRT analyses. This type of test design that utilizes common items for linking purposes is widely used in practice (von Davier 2011).

### 8.4. Data Analysis

The conventional scores (i.e., sum score or proportion correct) are straightforward and easy to understand. However, these scores are not comparable across the five tests without assuming that each test is strictly parallel. That is, only tests that consist of *exactly* the same level of item characteristics (i.e., item difficulties and discriminations), which is unrealistic, can make the sum score and the percentage scores comparable across different tests.

To overcome this limitation, measurement models based on the IRT have been widely used in the field of educational measurement. The use of IRT models allows the resulting scores (often referred to as “latent abilities”, “skills”, or “theta”) to be comparable regardless of the items or test forms administered if the tests are designed to facilitate it, as shown in the example above (Table 4).

For the data collected through the common item set design explained above, we have applied the IRT models to the five assessments data altogether. Treating and analyzing the data in this way presumes that the item parameters are invariant (i.e., a difficult item is difficult regardless of the assessment), whereas the change in score can be attributed to the student’s growth or change. Specifically, the Generalised Partial Credit Model (GPCM; (Muraki 1992)), which estimates the discrimination and difficulty for each item, was fitted. The GPCM is written as follows:

$$P_{jk}\left(\theta \right)=\frac{\exp\left[\sum_{v=0}^{k}a_{j}\left(\theta -b_{jv}\right)\right]}{\sum_{c=0}^{4}\exp\left[\sum_{v=0}^{c}a_{j}\left(\theta -b_{jv}\right)\right]}$$

where $P_{jk}$ is the probability of obtaining the score of *k* (*k* = 0, 1, 2, 3, 4) of an item *j* given that the student’s latent critical thinking skill is θ, $a_{j}$ is discrimination parameter, and $b_{jv}$ are step parameters that can vary across items.

The resulting scores, estimated as weighted likelihood estimates (WLE; (Warm 1989)) from the GPCM, are on the logit scale, which ranges from negative to positive infinity. As negative scores are not intuitively interpretable for practitioners, it is common to transform logit scores to a more familiar positive scale. To facilitate the interpretation of the scores, the *PACIER scale scores* were computed through a linear transformation by converting the logit scores resulting from the IRT models. The PACIER scale scores of the Year 6 students were designed to have a mean of 100 and a standard deviation of 10 and were reported to the schools.

## 9. Psychometric Properties of the PACIER Critical Thinking Assessment

### 9.1. Results of IRT Analyses

When the GPCM was fit to the data comprising 96 items collected from three schools across five assessments, 2 items were estimated to have negative or near-zero slopes. Both items were developed to measure the Creative Thinking of the PACIER domains. Because those 2 items did not contribute to estimating the proficiency, they were excluded from the further analyses.

**Figure 2:**
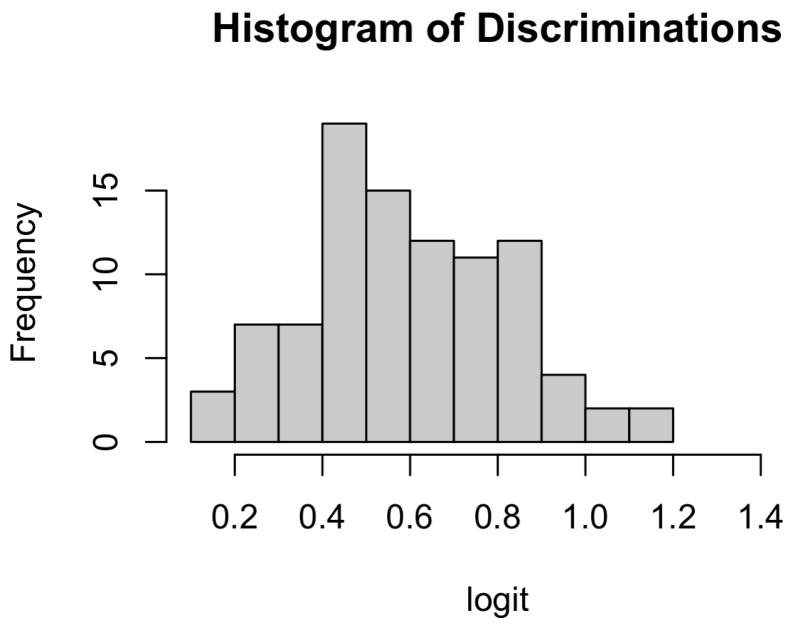
Histogram of Discrimination Parameter Estimates.

Figure 2 illustrates the distribution of item discrimination parameter estimates. After excluding 2 Creative Thinking items, the rest of the 94 items behaved well, showing the range of discriminations from 0.16 to 1.12 with the median of 0.58. Although not presented, the step parameters are within the range of -6 to 6 logit that are normally observed in testing programs. Overall, except for 2 items, discrimination and difficulty item parameters appeared to be within the acceptable range.

**Figure 3:**
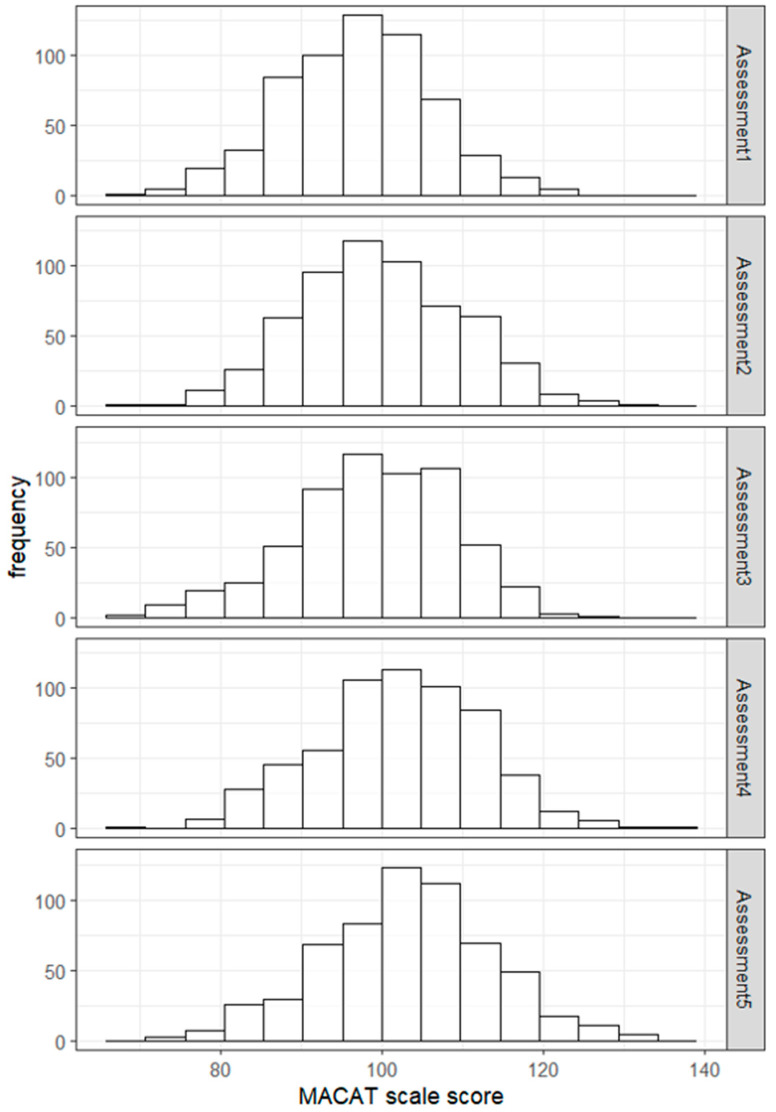
Histograms of students’ performance per assessment.

Assuming that item parameters were estimated satisfactorily, the performance of students was calculated and transformed to the PACIER scale score (Figure 3, Table 5). Considering that the PACIER scale scores were constructed to have a general mean of 100 and SD of 10, there was a slight increase in the overall performance, as much as 5 points on the PACIER scale, from the first assessment to the fifth assessment.

### 9.2. Reliability

The marginal IRT reliability returned after the GPCM was estimated to be 0.953. This means that the 94-item PACIER Critical Thinking Assessment, taken as a whole, can measure critical thinking skills with a high degree of reliability. Given that this reliability does not take into account the interdependencies between five tests for the same student, the reliability of the PACIER Critical Thinking Assessment was estimated separately for each assessment. They were also all highly acceptable: 0.845 for the first assessment, 0.884 for the second, 0.934 for the third, 0.966 for the fourth, and 0.906 for the fifth.

### 9.3. Validity

The AERA (American Educational Research Association 2014) Standards outline five sources of validity evidence: test content, response processes, internal structure, relations to other variables, and consequences of testing. Below, in terms of test content, convergent and discriminant validity were examined using the multidimensional IRT model to investigate the structure exhibited by the PACIER Critical Thinking Assessment. Convergent and discriminant validity suggest that measures of the same construct should be highly intercorrelated among themselves, while cross-construct correlations should be at a lower level than the within-construct correlations.

To do this, an extended GPCM that maps each item to one of the PACIER domains was analyzed. Because each item was designed to measure solely one single domain of the PACIER domains, it can be viewed as the between-multidimensional model or confirmatory IRT model (Adams et al. 1997). The result of primary interest of this analysis is the correlation structure among six PACIER domains. Table 6 presents the latent correlations among proficiency estimates across PACIER domains. The strongest correlations were observed between evaluation, reasoning, and interpretation at about 0.46, while the weakest correlation was observed between problem solving, creative thinking, and reasoning at about 0.35. According to Brown (2015), the correlation between constructs should be less than 0.80 for the discriminant validity. Given that the inter-skill correlations are all between 0.35 and 0.46, it supports the empirical evidence that PACIER domains are somewhat distinctive to each other, but at the same time, moderately correlated towards measuring critical thinking skills as a whole.

For the convergent validity, Brown (2015) suggested that standardized factor loadings should be greater than 0.3. As summarized in Table 7, all standardized factor loadings exceeded 0.3. Taken together, convergent and discriminant validity of the PACIER Critical Thinking Assessment was empirically supported.

Finally, the internal structure was examined using the approach described by Wilson (2023). Specifically, the average individual proficiency estimate (WLE) was computed for each score category per item. It is expected that the average WLE for the higher score category would be higher because higher performing students are expected to score higher on individual items. As expected, the difference in WLE from the higher score category to the adjacent lower score category turned out to be negative in only 11 cases out of a total of 375 (0.03%), while the rest showed positive values. Overall, this provides further evidence of the validity of the internal structure of the PACIER Critical Thinking Assessment.

## 10. Discussion and Conclusions

The PACIER framework offers a structured approach to conceptualizing and measuring critical thinking. It provides a theoretical framework inspired by the existing research literature and is translated into a cognitive assessment. The test items show adequate measurement properties, based on a pilot study with middle school students at an international school in Dubai. The PACIER Critical Thinking Assessment offers the possibility to evaluate each component of the PACIER framework—problem solving, analysis, creativity, interpretation, evaluation, and reasoning. The test items in the assessment were carefully and thoroughly developed by human experts to reflect the PACIER framework’s measurement construct. The design of the PACIER Critical Thinking Assessment that utilizes the item bank for targeting each grade level allows a subset of items to be selected and used at each testing occasion. Thus, it allows researchers and practitioners to examine students’ growth in critical thinking over time without using the same items. In addition, it is meaningful to use the overall score as an indicator of general critical thinking ability because the subcomponents are positively intercorrelated. The results of the empirical analyses from the pilot test showed a satisfactory level of psychometric properties and supported the acceptable reliability and validity (i.e., convergent and discriminant validity, internal structure) of the PACIER Critical Thinking Assessment.

In the future, it would be worthwhile to examine the stability of the measurement with a new sample to see if the PACIER framework can be generalized across cultures and languages. This is because measuring critical thinking skills, a core 21st-century skill, according to a well-established framework, will be important for innovating fundamental curricula and learning materials worldwide. Furthermore, developing innovative item types would improve the reliability, validity, and comparability of the PACIER Critical Thinking Assessments. Currently, test items consist of multiple-choice, fill-in-the-blank, and drag-and-drop types, which may not ideally measure higher-order, complex skills. Finally, the use of artificial intelligence (AI) for automatically generating and scoring critical thinking skills can be studied further. Preliminary studies have demonstrated the potential of machine-generated items (e.g., (Kim et al. 2025; Shin et al. 2024)), but the collaboration with humans in developing assessment tools and generating valid and reliable learning materials remains to be explored.

In terms of further research directions to be explored, it would be worthwhile to examine the links between PACIER skills, metacognition, self-regulation and executive functions. In particular, we would expect that metacognition and self-regulation are particularly solicited in problem solving and creative thinking components, whereas executive functions are most relevant to evaluation, reasoning, interpretation and analysis components.

Lastly, in addition to the PACIER Critical Thinking Assessment, there are PACIER-based training activities that are designed to help students engage with each component of critical thinking in classroom lessons. This curriculum for middle school students and university students will be described in a forthcoming report. In general, the PACIER curriculum explains the skills needed and proposes target activities to develop each. Example activities are as follows: (1) Problem Solving—develop a multistep plan to resolve a social challenge like traffic congestion; (2) Analysis—compare two data graphs on a topic like climate change and analyze what each one reveals or hides; (3) Creative Thinking—engage in “what if” thinking to explore multiple alternative ideas; (4) Interpretation—examine a text, such as a poem, and note different interpretations based on the tone and diction when the poem is read; (5) Evaluation—rank alternative decisions on criteria such as ethical issues or long-term consequences; (6) Reasoning—map out the logic of an argument as it develops in a documentary video. Linked to the PACIER educational activities, the PACIER Critical Thinking Assessment, with its IRT item characteristics, offers an opportunity to see training effects over time and to monitor more specifically the impact on particular critical thinking components, as designated in the PACIER framework.

## Figures and Tables

**Table 1:** PACIER model.

	Problem Solving	Analysis	Creative Thinking	Interpretation	Evaluation	Reasoning
SKILLS	Producing strong solutions	Understanding how an argument is built	Creating new connections and unexpected solutions	Looking at issues of meaning	Exploring strengths and weaknesses of an argument	Creating strong and persuasive arguments
SUB-SKILLS	Asking productive questions	Working out the functions of each part of an argument	Connecting things together in a new way	Seeking to clarify meaning where necessary	Judging the acceptability of the reason(s) used in terms of readability	Producing well structured arguments
	Generating possibilities	Understanding the relationships between parts of an argument	Producing novel explanations for existing evidence	Grasping the meaning of technical terms	Judging the relevance of the reason(s) used	Dealing with counter-arguments
	Generating solutions	Showing the structure of an argument	Generating new hypotheses	Understanding the meaning of available evidence	Judging the adequacy of the reason(s) used	Evaluating the reasoning of arguments
	Making sound decisions	Looking for assumptions in an argument	Redefining an issue so as to see it in a new way	Highlighting problems of definition	Judging what would strengthen or weaken an argument	Looking at the need to persuade

**Table 2:** Sample Items PACIER.

***PROBLEM SOLVING*** Marine life adapts to the conditions of the ocean. In the deep sea, there is little light, yet the anglerfish thrive. Question: Choose the questions to find out how an anglerfish survives in the deep sea. Click on: D if the question will definitely help. P if the question may possibly help. N if the question will not help. How many species of anglerfish exist? D P N (Answer: N) Why does an anglerfish have a bioluminescent lure? D P N (Answer: D) Does the pressure in the deep sea affect the anglerfish’s body structure? D P N (Answer: D) What do anglerfish eat in the deep sea? D P N (Answer: P)
***ANALYSIS*** Centuries ago, the Aztecs constructed some of the earliest pyramids using clay and stone, situated on elevated platforms surrounded by canals. Over time, the Aztecs enhanced these pyramids with intricate carvings and vibrant colors—many of these remarkable structures can still be seen today. The pyramids were designed to honor their gods but also served as places for ceremonies and gatherings for the community. Question: Here are four statements. Read the statement and decide if it is true or not true of the information in the text. Click ‘True’ if the statement is true of the information in the text. Click ‘Not true’ if the statement is not true of the information in the text. The text is written by someone who is knowledgeable about ancient civilizations. True Not true (Answer: True) The purpose of the text is to convince people that the Aztecs were extraordinary. True Not true (Answer: Not true) The author appreciates the craftsmanship of the Aztec pyramid builders. True Not true (Answer: True) The author believes that pyramids were central to the Aztec community. True Not true (Answer: True)
***CREATIVE THINKING*** We often hear that exercise is crucial for maintaining good health. For adults, this appears to be accurate. Numerous studies show that adults who engage in regular exercise tend to have better overall health than those who do not. Question: Here are four statements that might explain the connection between regular exercise and improved health. Click on: ‘Yes’ if the statement could explain the connection. ‘No’ if the statement could not explain the connection. Adults who exercise regularly usually have more free time than those who do not. Yes No (Answer: No) Exercise boosts energy levels in adults. Yes No (Answer: Yes) Engaging in regular exercise enhances mental well-being. Yes No (Answer: Yes) People choose different types of exercise routines. Yes No (Answer: No)
***INTERPRETATION*** Installing green walls on urban buildings helps reduce noise pollution. Researchers compared various plant species and found a dense-leaved ivy to be the most effective. The ivy absorbs sound waves from traffic noise. Each square meter of ivy can reduce noise levels equivalent to a 10-decibel decrease. Question: Read the information to say whether the statements are known for certain or not. Click ‘Known’ if a statement is known for certain from the information. Click ‘Not known’ if a statement is not known for certain from the information. Researchers know how much noise a square meter of ivy can reduce. Known Not known (Answer: Known) The dense leaves of the ivy absorb sound waves. Known Not known (Answer: Not known) The ivy is not the only plant species to have been studied. Known Not known (Answer: Known) Trees also help reduce noise pollution. Known Not known (Answer: Not known)
***EVALUATION*** Jordan says, ‘Teenagers should be allowed to watch TV shows.’ Taylor says, ‘It is not beneficial for teenagers to watch TV shows—they should be engaging in physical activities, reading books, or exploring the outdoors.’ Question: Below are four statements. If you think a statement supports teenagers watching TV shows, choose ‘Supports’. If you think a statement does not support teenagers watching TV shows, choose ‘Does not support’. Watching TV shows can help teenagers unwind. Supports Does not support (Answer: Supports) Teenagers who watch a lot of TV shows tend to have less physical activity. Supports Does not support (Answer: Does not support) Experts believe watching TV shows can improve language skills. Supports Does not support (Answer: Supports) If teenagers spend time watching TV shows, they miss out on social interactions. Supports Does not support (Answer: Does not support)
***REASONING*** Lila wants to convince her parents that she should be allowed to have a pet dog. The sentences have some words missing. Question: Choose the correct words from the drop-down list to complete the text. Use four of the five words. _______ believed to be a lot of work, but we now know there are many benefits to having a pet dog. _______, studies have shown that having a dog can improve mental health and encourage physical activity. ________, regular walks with a dog can help maintain a healthy lifestyle. _________ families with dogs might experience more happiness and bonding. Options: Dogs were Rarely Consequently Additionally For instance *Correct answers in the following order: Dogs were, For instance, Additionally, Consequently*

**Table 3:** Number of participating students per school per assessment for five test periods.

Schools	1st Test	2nd Test	3rd Test	4th Test	5th Test
School 1	276	321	330	335	318
School 2	125	167	166	119	119
School 3	138	198	199	195	190
Total number of students	539	686	695	649	627

Note: School 1 was GEMS Modern Academy, School 2 was the New Millennium School, School 3 was The Millennium School.

**Table 4:** Example of MACAT pilot test design.

Assessments	Clusters (96 Items in Total Across 8 Clusters)							
	A	B	C	D	E	F	G	H
1 (24 items)	12 items	12 items						
2 (24 items)				12 items	12 items			
3 (24 items)			12 items			12 items		
4 (24 items)	12 items						12 items	
5 (24 items)			12 items					12 items

**Table 5:** Mean and SD of students’ performance per assessment.

	Mean	SD
1	96.9	9.2
2	99.9	9.9
3	99.5	10.2
4	101.7	10.0
5	102.2	10.0

**Table 6:** Correlations of multidimensional PACIER proficiency estimates.

	P	A	C	I	E	R
P	1.000					
A	0.369	1.000				
C	0.352	0.364	1.000			
I	0.375	0.445	0.389	1.000		
E	0.373	0.399	0.421	0.459	1.000	
R	0.355	0.451	0.414	0.457	0.467	1.000

**Table 7:** Distribution of standardized factor loadings for PACIER domains.

	P	A	C	I	E	R
N of items	16	16	14	16	16	16
min	0.375	0.415	0.447	0.440	0.434	0.458
median	0.466	0.600	0.589	0.598	0.488	0.506
max	0.512	0.687	0.664	0.725	0.552	0.621

## Data Availability

Data available on request, restricted access.
